# Dependence of the content of starch and reducing sugars on the level of expression of the genes of β-amylases StBAM1 and StBAM9 and the amylase inhibitor StAI during long-term low-temperature storage of potato tubers

**DOI:** 10.18699/VJGB-22-62

**Published:** 2022-10

**Authors:** A.V. Kulakova, G.I. Efremov, A.V. Shchennikova, E.Z. Kochieva

**Affiliations:** Institute of Bioengineering, Federal Research Centre “Fundamentals of Biotechnology” of the Russian Academy of Sciences, Moscow, Russia; Institute of Bioengineering, Federal Research Centre “Fundamentals of Biotechnology” of the Russian Academy of Sciences, Moscow, Russia; Institute of Bioengineering, Federal Research Centre “Fundamentals of Biotechnology” of the Russian Academy of Sciences, Moscow, Russia; Institute of Bioengineering, Federal Research Centre “Fundamentals of Biotechnology” of the Russian Academy of Sciences, Moscow, Russia

**Keywords:** Solanum tuberosum, potato cultivars, tuber storage, starch catabolism, gene expression, β-amylase, Solanum tuberosum, сорта картофеля, хранение клубней, катаболизм крахмала, экспрессия гена, β-амилаза

## Abstract

Solanum tuberosum L. is the most important non-grain starch crop with a potential yield of 38–48 t/ha and a starch content of 13.2–18.7 %. Potato tubers are stored at a low temperature (2–4 °C) in a state of physiological dormancy. A disadvantage of this type of storage is the degradation of starch and the accumulation of reducing sugars (cold-induced sweetening), including due to an increase in the activity of β-amylases that hydrolyze starch to maltose. In this study, a comparative analysis of the β-amylase (StBAM1, StBAM9) and amylase inhibitor (StAI ) gene expression, as well as starch and reducing sugar content in tubers during long-term low-temperature storage (September, February, April) was performed using potato cultivars Nadezhda, Barin, Krasavchik, Severnoe siyanie and Utro. The β-amylase genes, StBAM9 and one of the two StBAM1 homologs (with the highest degree of homology with AtBAM1), were selected based on phylogenetic analysis data. Evaluation of the expression of these genes and the amylase inhibitor gene showed a tendency to decrease in transcription for all analyzed cultivars. The starch content also significantly decreased during tuber storage. The amount of reducing sugars increased in the September–April period, while in February–April, their content did not change (Krasavchik), decreased (Barin, Severnoe siyanie) or continued to grow (Utro, Nadezhda). It can be assumed that the gene activity of StBAM1 and StBAM9 correlates with the amount of starch (positively) and monosaccharides (negatively). The level of StAI expression, in turn, may be directly dependent on the level of StBAM1 expression. At the same time, there is no relationship between the degree of cultivar predisposition to cold-induced sweetening and the expression profile of the StBAM1, StBAM9, and StAI genes.

## Abstract (ru)

Картофель (Solanum tuberosum L.) – первая по важности незерновая крахмалоносная культура с уровнем потенциальной урожайности 38–48 т/га и содержанием крахмала 13.2–18.7 %. Клубни картофеля хранятся при низкой температуре (2–4 °С), что обеспечивает состояние физиологического покоя. Недостатком такого хранения являются распад крахмала и, как следствие, накопление редуцирующих сахаров (холодовое осахаривание), в том числе за счет роста активности β-амилаз, гидролизующих крахмал до мальтозы. В настоящем исследовании проведен сравнительный анализ динамики экспрессии генов β-амилаз (StBAM1, StBAM9) и ингибитора амилаз (StAI), а также содержания крахмала и редуцирующих сахаров в процессе длительного низкотемпературного хранения (сентябрь, февраль, апрель) клубней пяти сортов картофеля (Надежда, Барин, Красавчик, Утро и Северное сияние). Гены β-амилаз – StBAM9 и один из двух гомологов StBAM1 (с наибольшей степенью гомологии с AtBAM1) – выбраны на основе данных филогенетического анализа. Оценка экспрессии этих генов, а также гена ингибитора амилаз показала тенденцию к снижению уровня транскрипции для всех анализируемых сортов. Обнаружено, что содержание крахмала в процессе хранения клубней также существенно падает. В то же время количество редуцирующих сахаров увеличивается в период сентябрь–апрель, тогда как в период февраль–апрель их содержание не меняется (Красавчик), снижа- ется (Барин, Северное сияние) или продолжает расти (Утро, Надежда). Можно предположить, что активность генов StBAM1 и StBAM9 коррелирует с количеством крахмала (положительно) и моносахаридов (отрица- тельно). А уровень экспрессии StAI, в свою очередь, находится в прямой зависимости от уровня экспрессии StBAM1. При этом зависимость между степенью предрасположенности сорта к холодовому осахариванию и профилем экспрессии генов StBAM1, StBAM9 и StAI отсутствует.

## Introduction

Starch is a polymer of glucose and is one of the three main natural polysaccharides. Unlike cellulose and chitin (structural biopolymers of the cell), starch is the main storage carbohydrate and is found in large quantities in plastids of heterotrophic plant organs: tubers and roots (tuber and root crops), grains (cereals and legumes), mature and/or immature fruits (Benkeblia et al., 2008; Bello-Perez et al., 2020).

The presence of starch in tubers of potato (Solanum tuberosum L.), the fourth most important crop in the world (after cereals), determines its universal use as a food, fodder and industrial crop. Despite the fact that cultivated cereals also have a high content of this polysaccharide in grains, the advantage of using potato starch is provided by its physicochemical properties (granule structure, physicochemical properties, the ratio of amylose and amylopectin polysaccharides, the degree of polymerization of molecules, etc.). Potato cultivars differ in the amount of starch in tubers, but varieties with almost any starch content and characteristics are eaten, determining the choice of a cooking method, as well as digestibility and glycemic response (Bello-Perez et al., 2020).

The content of starch in tubers is determined primarily by the genetic component, namely, the activity of more than 70 genes, including genes for key enzymes of biosynthesis (starch synthase, etc.) and degradation (starch phosphorylase, adenylate kinase, amylases, etc.) (Van Harsselaar et al., 2017). Also, the amount of polysaccharide is affected by post-harvest storage of tubers at low positive temperatures (2–4 °C). Thus, a state of physiological dormancy is maintained, while germination, drying out and development of infections are slowed down. At the same time, by the end of the storage period (closer to the planting season), part of the starch is degraded with the formation of glucose, which is necessary to stimulate the growth of shoots (Benkeblia et al., 2008). However, a number of varieties are characterized by the cold-induced sweetening (CIS) – a significant increase in the content of reducing sugars in response to low temperatures (Fischer et al., 2013), which leads to a deterioration in nutritional and dietary qualities, in particular due to the formation of acrylamide during frying (Sonnewald S., Sonnewald U., 2014; Hou et al., 2019; Tai et al., 2020). At the same time, there are CIS-resistant varieties that are used for the production of french fries.

Starch catabolism is important both for plant growth and in terms of consumer properties. The degree of susceptibility of starch to degradation depends on the composition and structure of the granules, which determines the digestibility of starch and the glycemic response (Bello-Perez et al., 2020). Under the action of α-glucan water dikinase (GWD; EC 2.7.9.4) and phosphoglucan, water dikinase (PWD; EC 2.7.9.5), starch is degraded into branched and linear glucans (Fettke et al., 2007; Shoaib et al., 2021). Degradation to oligosaccharides and maltose molecules is catalyzed by phosphorolytic (starch phosphorylases, EC 2.4.1.1) and hydrolytic (α-amylases, or 1,4-α-D-glucan-glucanohydrolases, AMY, EC 3.2.1.1; β-amylases, or 1,4-α-D-glucan maltohydrolases, BAM or Bmy, EC 3.2.1.2) enzymes (Solomos, Mattoo, 2005; Zeeman et al., 2007; Shoaib et al., 2021). AMY hydrolyzes endo-α- 1,4-glycosidic bonds, forming oligosaccharides of various lengths, while BAM cleaves off the second from the end α-1,4-glycosidic bond, releasing disaccharides (Zeeman et al., 2007; Shoaib et al., 2021). The release of glucose molecules occurs under the exo-action of α-glucosidases (1,4-α-d-glycan- glucohydrolase, EC 3.2.1.20), which break the extreme α-1,4- and α-1,6-glycosidic bonds (Taylor et al., 2000). The reduced activity of both α-amylases and α-glucosidases significantly reduces the rate of starch hydrolysis, which is a positive effect both for preventing CIS of tubers during storage and for increasing the dietary value of potato (Riyaphan et al., 2018).

According to studies of β-amylases in various plant species, these hydrolases are also highly significant for starch hydrolysis. In the model species Arabidopsis thaliana L., a family of β-amylases is characterized, consisting of nine enzymes with different localization and function (Monroe, Storm, 2018). Phylogenetic analysis of the amino acid sequences of β-amylases from 136 different species of algae and land plants showed that modern angiosperms contain eight clades of β-amylases, as well as a clade of inactive BAM10 enzymes, which is absent in Arabidopsis (Thalmann et al., 2019). At the same time, Arabidopsis BAM4 homologs are absent in many starchy crops, which suggests species-specific regulation of starch digestion (Thalmann et al., 2019).

The functional activity of individual enzymes of the BAM family is elucidated using various approaches and methods. Thus, the importance of the expression level of endospermspecific β-amylase (Bmy1) and constitutive Bmy2 genes during the development of barley grain for determining the quality of malting is demonstrated (Vinje et al., 2019). A significant role of the PbrBAM3 gene (birch pear Pyrus betulaefolia Bunge) in plant resistance to cold due to an increase in the level of soluble sugars is shown (Zhao et al., 2019). Most of the studies (mainly from the 1990s) are published on β-amylases of sweet potato (Ipomoea batatas (L.) Lam.), the results of which indicate the importance of this enzyme in modulating the properties of sweet potato starch in order to increase consumer qualities (Guo et al., 2019).

Despite the participation of β-amylases in the breakdown of starch shown in other plants, there are few works on their study in potato. It has been shown that these enzymes are capable of hydrolyzing potato tuber amylose to maltose without residue (Hopkins et al., 1948). The activity of β-amylases increases significantly when the storage temperature of tubers decreases from 20 °C to 3–5 °C (Nielsen et al., 1997), as well as during the germination of tubers at the physiological dormancy release (Vajravijayan et al., 2018). Transcriptomic and proteomic analysis of potato tubers stored at 15, 4, and 0 °C confirmed that the regulation of reducing sugar accumulation is positively associated with the expression of β-amylases (Lin et al., 2019).

The level of StBAM1 and StBAM9 gene expression positively correlates with the accumulation of reducing sugars in tubers stored at low temperatures (Zhang et al., 2014a). The StBAM1 enzyme can be inactivated by interaction with the amylase inhibitor SbAI (Zhang et al., 2014b), as well as by ubiquitination and degradation of StBAM1 triggered by the transcription factor SbRFP1 (Zhang et al., 2019).

In this study, the dynamics of the expression of genes for β-amylases StBAM1, StBAM9 and StAI amylase inhibitor, as well as changes in the content of starch and reducing sugars were analyzed in tubers of five potato cultivars (Nadezhda, Barin, Krasavchik, Utro, Severnoe siyanie) under long-term low-temperature storage. The choice of cultivars was due to differences in the tuber starch content.

## Materials and methods

In the study, tubers of five potato cultivars (Nadezhda, Barin, Krasavchik, Utro, Severnoe siyanie) were used, differing, according to the originators (https://reestr.gossortrf.ru/), in tuber starch content and purpose (Table 1). The plants were grown in 2021 in the field of the Federal Potato Research Center named after A.G. Lorch (Moscow region, Russia); at the end of August, two plants of each cultivar were transferred to the conditions of the experimental climate control facility in the Institute of Bioengineering (Research Center of Biotechnology, Russian Academy of Sciences). In September 2021, the tubers were collected, homogenized and used (peel and pulp together) for subsequent analysis of β-amylase (StBAM1 and StBAM9) and amylase inhibitor (StAI ) gene expression, as well as for determining the content of starch and reducing sugars (glucose and fructose).

**Table 1.**
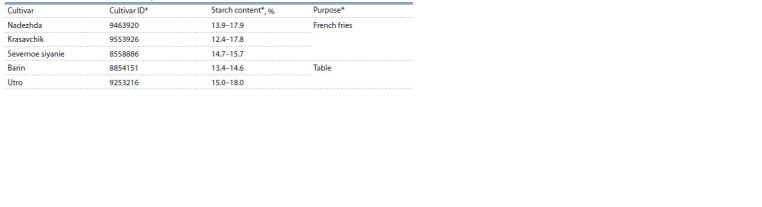
Potato cultivars used in the study According to: https://reestr.gossortrf.ru/.

Total RNA was isolated from 50–100 mg of tuber tissue (RNeasy Plant Mini Kit, QIAGEN, Germany), additionally purified from DNA impurities (RNase free DNasy set, QIAGEN) and used for cDNA synthesis (GoScript™ Reverse Transcription System, Promega, USA), according to manufacturer’s protocols. The quality of RNA was checked by electrophoresis in 1.5 % agarose gel. RNA and cDNA concentrations were determined on a Qubit 4 fluorimeter (Thermo Fisher Scientific, USA) using appropriate reagents (Qubit RNA HS Assay Kit and Qubit DS DNA HS Assay Kit, Invitrogen, USA).

Expression of the StBAM1, StBAM9, and StAI genes in potato tubers was analyzed by quantitative real-time PCR (qRT-PCR) with normalization of data using the reference genes elongation factor 1-alpha (elf1; LOC102600998) and SEC3A (LOC102599118) (Lopez-Pardo et al., 2013; Tang et al., 2017) (Table 2). For qRT-PCR, we used 3 ng of the cDNA template, cDNA-specific primers (see Table 2), the Reaction Mixture for RT-PCR in the Presence of SYBR GreenI and ROX kit (Sintol, Russia), and a thermal cycler CFX96 Real- Time PCR Detection System (Bio-Rad Laboratories, USA). The reactions were carried out in two biological and three technical replicates under the following conditions: 5 min at 9 °C, 40 cycles (15 s at 95 °C, 50 s at 62 °C).

**Table 2.**
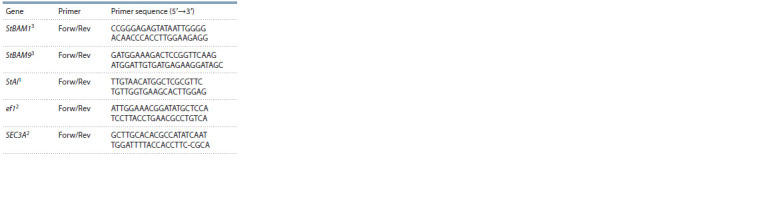
Primers used for qRT-PCR 1 Dyachenko et al., 2021; 2 Lopez-Pardo et al., 2013; Tang et al., 2017; 3 primers developed in this study.

The β-amylase gene sequences from S. tuberosum (BAM1, gene ID 102598794; BAM1, 102584887; BAM8, 102598339; PCT-BMY1, 102577806; BAM3, 102594291; BAM-like, 102584563; BAM7, 102593066; BAM9, 102590483) and model species A. thaliana (BAM1, 821975; BAM2, At2g45880, 827959; BMY2 (BAM8), 834566; CT-BMY (BAM3), 827419; BAM4, AT5G55700, 835664; BAM5, 827185; BAM6, 817789; BAM7, AT2G45880, 819196; BAM9 (BMY3), At5g18670, 831985) were obtained from the NCBI database (https://www. ncbi.nlm.nih.gov). The phylogeny of the β-amylase protein sequences was assessed to determine the S. tuberosum homologs of Arabidopsis β-amylases, which are most significant in starch degradation. The analysis was performed with MEGA7 (https://www.megasoftware.net/) using the maximum likelihood method based on the JTT model; bootstrap – 1000 replicates. Based on the transcripts of the S. tuberosum β-amylase genes, we designed primers for the analysis of StBAM1 (gene ID 102584887) and StBAM9 (gene ID 102590483) expression (see Table 2); the forward and reverse primers were separated by at least one intron. The gene specificity of primers was verified by comparing their sequences with S. tuberosum transcripts using the NCBI-primer-blast (https://www.ncbi. nlm.nih.gov/tools/primer-blast/).

Starch content (mg/g fresh tissue) was determined using an Eppendorf BioSpectrometer® basic (Eppendorf, Germany; λ = 340 nm) and a Starch enzyme test (Boehringer Mannheim/ R-Biopharm AG, Switzerland) with some modifications to the manufacturer’s protocol.

Briefly, tuber material (together pulp and peel) (~0.02 g; this amount was determined based on known data on the average starch content in potato tubers (13–20 %) and test requirements for the amount of starch in the sample) was homogenized, suspended in the mixture of 1 ml dimethyl sulfoxide (DMSO) and 0.25 ml concentrated hydrochloric acid, and incubated at 60 °C for 60 min with shaking. Then it was cooled to 25 °C, mixed with 2.5 ml of milliQ; the pH was adjusted to 4.5 with 2N sodium hydroxide. The suspension was settled or filtered through Miracloth (Merck, USA). An aliquot of the supernatant was diluted 5, 10, 20 and 100 times; 0.05 ml of the resulting solution was used for the enzyme test and subsequent spectrophotometry. The values corresponding to ΔA = 0.115 ± 0.035 were considered (based on the manufacturer’s recommendations). The analysis was carried out in two biological and three technical replicates.

The content of reducing sugars (glucose and fructose) (mg/g fresh tissue) was measured using high performance liquid chromatography (HPLC) with a Varian ProStar chromatograph (Varian Inc., USA), a 102 M differential refractive index detector for the chromatograph (Stayer model, Khromatek, Russia) and Agilent Pursuit 200Å PFP columns (4.6 × 150 mm, 5 μm HPLC Column, A3050150X046, Agilent, USA). Briefly, 1 g of the tuber material (together the pulp and peel) was ground in liquid nitrogen, suspended in 10 ml of 80 % ethanol, and centrifuged at 16,000 g for 15 min. The supernatant was used for HPLC analysis. Isocratic elution was performed with acetonitrile: water (75:25 v/v) as the mobile phase; flow rate – 1.5 ml/min, temperature – 30 °C. The analysis was carried out in two biological and three technical replicates

Statistical processing of the qRT-PCR and the starch and sugar content data was performed using the GraphPad Prism v. 8 (GraphPad Software Inc., USA; https://www.graphpad.com/ scientific-software/prism/). Data were expressed as mean (M) with standard deviation (± SD) based on two biological and three technical replicates for each measurement option. Welch’s t-test (unequal variance) was used to assess differences in gene expression and carbohydrate content ( p < 0.05 indicates statistical significance of differences).

## Results

**Fig. 1.**
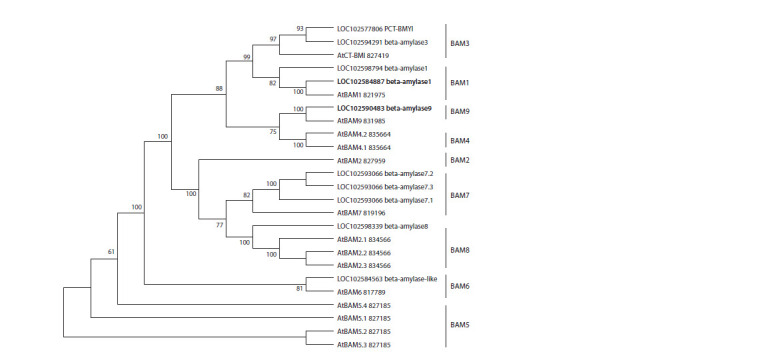
Unrooted consensus dendrogram based on the alignment of 25 amino acid sequences of β-amylases from S. tuberosum (BAM1, gene ID 102598794; BAM1, 102584887; BAM8, 102598339; PCT-BMY1, 102577806; BAM3, 102594291; BAM-like, 102584563; BAM7, 102593066; BAM9, 102590483, including isoforms) and model species A. thaliana (BAM1, 821975; At2g45880 BAM2, 827959; BMY2 (BAM8), 834566; CT-BMY (BAM3), 827419; AT5G55700 BAM4, 835664; BAM5, 827185; BAM6, 817789; AT2G45880 BAM7, 819196; At5g18670 BAM9 (BMY3), including isoforms). The analysis was carried out in the MEGA 7.0 using the maximum likelihood method based on the JTT model. Branches corresponding to clusters replicated in less than 50 % of bootstrap replicates are collapsed. The percentage of repeating trees where related taxa are grouped together in the bootstrap test (1000 replicates) is shown next to the branches.

The study was focused on the characterization of the expression of three genes – StBAM1, StBAM9 and StAI. The amylase inhibitor gene (StAI, gene ID 102591697) is present in the potato genome in one copy (Zhang et al., 2014b; Dyachenko et al., 2021), while the β-amylase family consists of several members (Van Harsselaar et al., 2017). Based on the available NCBI and published data, the available sequences of the S. tuberosum and A. thaliana β-amylase genes were obtained. Comparative structural-phylogenetic analysis of the encoded enzymes classified S. tuberosum β-amylases according to their homology with A. thaliana proteins that form nine clades (AtBAM1–AtBAM9) (Fig. 1).

S. tuberosum homologs were found for seven clades of A. thaliana β-amylases (with the exception of AtBAM2 and AtBAM4). In particular, two StBAM1 and one StBAM9 (gene ID 102590483) homologs were identified in the potato genome. Based on the obtained dendrogram, StBAM1 (gene ID 102584887) with the highest degree of homology with AtBAM1 was selected from two β-amylases of the BAM1 clade for study (see Fig. 1). We designed primers (see Table 2) for the selected genes StBAM1 (gene ID 102584887) and StBAM9 (gene ID 102590483) and used them to analyze their expression.

Tubers of five potato cultivars, Nadezhda, Krasavchik, Severnoe siyanie, Barin, Utro (see Table 1), were collected in September and stored at +3 °C. Tuber tissues were collected for expression and biochemical analyses in September (fresh harvest), February (5–6 months of storage) and April (8 months of storage).

**Fig. 2.**
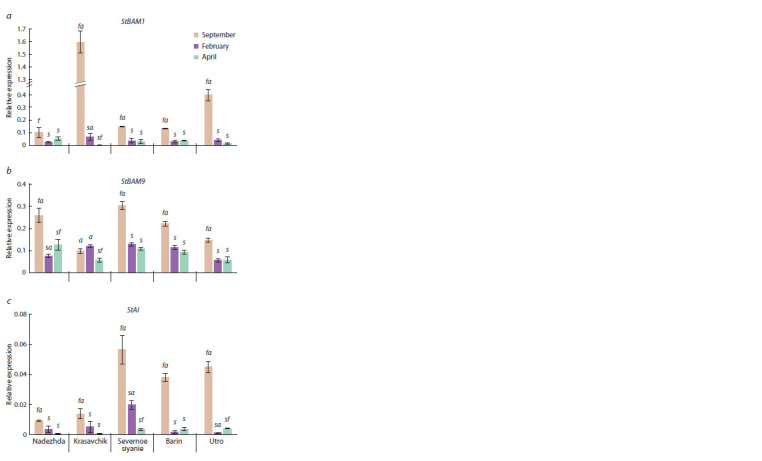
StBAM1 (a), StBAM9 (b), and StAI (c) gene expression pattern in tubers of f ive potato cultivars (Nadezhda, Krasavchik, Severnoe siyanie, Barin, Utro) during low-temperature (+3 °C) storage (September, February, April). The letters s, f, and a above the columns indicate a signif icant difference ( p < 0.05) of a particular value of gene expression from the values for two other months within each sample (s – September, f – February, a – April).

To determine the possible activity of StBAM1, StBAM9 and StAI, key enzymes of starch degradation (Zhang et al., 2014a, b), the expression of genes encoding them in tubers was analyzed during low-temperature storage (+3 °С; September, February, April) (Fig. 2). A significant decrease in StBAM1 expression was shown in April compared to September (most pronounced in cv. Krasavchik and Utro). At the same time, the difference between the February and April data was insignificant: the level of gene expression continued to slightly decrease or did not change (see Fig. 2).

StBAM9 expression also decreased significantly in February compared to September, but not as sharply as StBAM1 expression. The exception was cv. Krasavchik, where the StBAM9 transcription has not changed. In April, compared to February, StBAM9 expression slightly increased (Nadezhda), did not change (Utro, Barin, Severnoe siyanie), or decreased (Krasavchik) (see Fig. 2).

A similar trend was also observed for the StAI gene. Its expression sharply decreased in April compared to September in cv. Severnoe siyanie, Barin and Utro. In cv. Nadezhda and Krasavchik, the level of StAI transcription decreased smoothly. In April, as compared to February, StAI expression slightly increased (Utro), did not change (Nadezhda, Krasavchik, and Barin), or sharply decreased (Severnoe siyanie) (see Fig. 2).

Thus, we observed a similar trend towards a decrease in the expression level for all three analyzed genes in potato tubers during low-temperature storage.

**Fig. 3.**
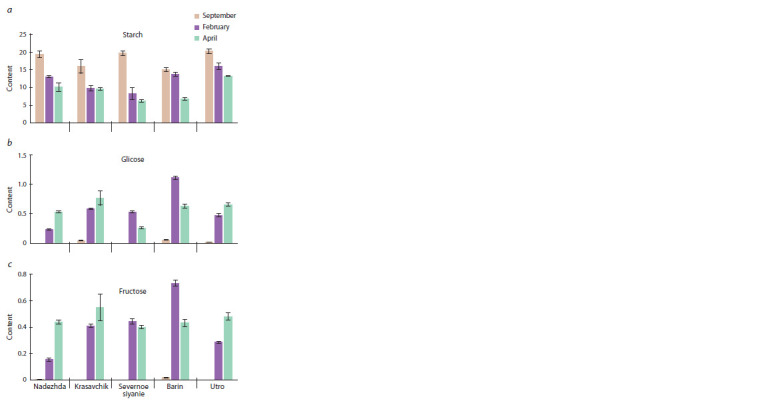
The content of starch and reducing sugars (glucose, fructose) (mg/g fresh tissue) in potato tubers of f ive cultivars (Nadezhda, Krasavchik, Severnoe siyanie, Barin, Utro) during low-temperature (+3 °C) storage (September, February, April).

To assess the possible correlations between the expression of β-amylase and amylase inhibitor genes with the content of starch and reducing sugars in the tubers, a biochemical analysis of the content of starch, glucose, and fructose was carried out during low-temperature storage of tubers (September, February, April) (Fig. 3).

As expected, compared to September, the starch content in tubers of all cultivars significantly decreased in April (see Fig. 3). At the same time, the content of reducing sugars in February and April in all cultivars was significantly higher than in September. Compared with February, in April the content of glucose and fructose in the tubers of cv. Utro, Nadezhda, and Krasavchik continued to grow, while in cv. Barin and Severnoe siyanie, it sharply decreased (see Fig. 3). At the same time, in February, the tubers of cv. Barin had the highest content of fructose and glucose – 1.5–3.0 and 1.5–4.0 times higher than in the other cultivars. The lowest rates were in the tubers of cv. Nadezhda. In April, there were no significant differences between the cultivars, except for a lower (compared to the other cultivars) glucose content in the cv. Severnoe siyanie tubers.

Thus, during low-temperature storage from September to April, the starch content in tubers of all cultivars decreased to varying degrees, and the content of reducing sugars increased in tubers of cv. Nadezhda and Utro. In cv. Krasavchik, Barin and Severnoe siyanie, the content of reducing sugars increased from September to February, and in April it did not change compared to February (Krasavchik) or significantly decreased (Barin, Severnoe siyanie).

## Discussion

Potato tubers stored at a low temperature (+3 °C) were characterized in dynamics (harvest, 5–6 and 8 months of storage) by the expression of β-amylase (StBAM1, StBAM9) and amylase inhibitor (StAI ) genes, as well as by the content of starch and reducing sugars. The five cultivars selected for analysis are divided into two groups depending on the purpose: table (Barin and Utro) and french fries (Nadezhda, Krasavchik, Severnoe siyanie) (see Table 1). This division is related to the degree of sensitivity of each cultivar to cold-induced sweetening of tubers; the higher the resistance, the more suitable the variety for the production of french fries, since in CIS-unstable varieties, frying is accompanied by an increased formation of reducing sugars, leading to the synthesis of acrylamide (Sonnewald S., Sonnewald U., 2014; Hou et al., 2019; Tai et al., 2020).

The accumulation of reducing sugars, which is characteristic of both cold-induced sweetening and the release of tubers from dormancy, positively correlates with the expression of β-amylase genes (Zhang et al., 2014a; Lin et al., 2019). The analyzed StBAM1 and StBAM9 are homologs of the A. thaliana BAM1 and BAM9 (see Fig. 1), localized in plastid and catalytically active (BAM1) or inactive (BAM9) (Monroe, Storm, 2018). The putative functional similarity of StBAM1 and StBAM9 with the corresponding A. thaliana enzymes is supported by other studies. Thus, it was shown that StBAM1 and StBAM9 make different contributions to the cold-induced sweetening of tubers. StBAM1 is localized in the amyloplast stroma and hydrolyzes soluble starch (Hou et al., 2017). StBAM9 is an inactive enzyme (Zhang et al., 2014b), but plays a dominant role in cold-induced sweetening (Hou et al., 2017). Localized on the surface of a starch granule, StBAM9 forms a protein complex with StBAM1, thus attracting catalytically active StBAM1 to release soluble glucan molecules from the surface of the granules (Hou et al., 2017). The StBAM1 enzyme can be inactivated by interaction with the amylase inhibitor SbAI (Zhang et al., 2014b), as well as by ubiquitination and degradation of StBAM1 triggered by the transcription factor SbRFP1 (Zhang et al., 2019).

Considering the above data, we expected an increase in the expression level of StBAM1 and StBAM9 and a decrease in StAI transcription in tubers during long-term low-temperature exposure (5–6 and 8 months). However, we observed a significant decrease in the expression of all three genes (see Fig. 2), although the starch content decreased and the amount of reducing sugars increased (see Fig. 3). It can be assumed that the StBAM1 and StBAM9 gene activity correlates with the amount of starch (positively) and monosaccharides (negatively). The level of StAI expression, in turn, may be directly dependent on the level of expression of the StBAM1 and α-amylase genes.

In addition, an increase in StBAM1 and StBAM9 expression was previously shown after 30 days of low temperature exposure (Zhang et al., 2014a), while in this study, analyses were performed 7 and 9 months after storage. We assume that after 30 days of storage of physiologically dormant tubers, it can be considered as a short-term cold stress, during which the tubers accumulate a sufficient amount of reducing sugars for cold resistance, after which an equilibrium is established between the content of starch/disaccharides and the activity of enzymes for starch degradation. In addition, the participation of α-amylases (hydrolysis) (Zhang et al., 2014a) and plastid starch phosphorylase (phosphorolysis) (Slugina et al., 2020) in starch catabolism should be taken into account.

## Conclusion

Considering the data obtained, it can be concluded that there is no relationship between the degree of cultivar predisposition to cold-induced sweetening of tubers and the expression profile of β-amylase (StBAM1, StBAM9) and amylase inhibitor (StAI ) genes.

## Conflict of interest

The authors declare no conflict of interest.
